# Community-acquired acute kidney injury in adults in Africa 

**DOI:** 10.5414/CNP86S121

**Published:** 2016-07-29

**Authors:** Dwomoa Adu, Perditer Okyere, Vincent Boima, Michael Matekole, Charlotte Osafo

**Affiliations:** School of Medicine and Dentistry, University of Ghana, Accra, Ghana

**Keywords:** acute kidney injury, Africa, infection, pregnancy, nephrotoxins

## Abstract

Aims: We review recent published data on demographics, causes, diagnoses, treatment, and outcome of acute kidney injury (AKI) in Africa. Methods: A review of the incidence, etiology, diagnoses, and treatment of AKI in adults in Africa from studies published between the years 2000 and 2015. Results: The incidence of AKI in hospitalized patients in Africa ranges from 0.3 to 1.9% in adults. Between 70 and 90% of cases of AKI are community acquired. Most patients with AKI are young with a weighted mean age of 41.3 standard deviation (SD) 9.3 years, and a male to female ratio of 1.2 : 1.0. Medical causes account for between 65 and 80% of causes of AKI. This is followed by obstetric causes in 5 – 27% of cases and surgical causes in 2 – 24% of cases. In the reported studies, between 17 and 94% of patients who needed dialysis received this. The mortality of AKI in adults in Africa ranged from 11.5 to 43.5%. Conclusions: Most reported cases of AKI in Africa originate in the community. The low incidence of hospital-acquired AKI is likely to be due to under ascertainment. Most patients with AKI in Africa are young and have a single precipitating cause. Prominent among these are infection, pregnancy complications and nephrotoxins. Early treatment can improve clinical outcomes.

## Introduction 

Recent studies show that minor acute changes in kidney function are associated with increased mortality [1, 2] and that acute kidney injury may be a harbinger of chronic kidney disease [3, 4]. There are many differences in the causes and treatment of cute kidney injury (AKI) between high- and middle-income countries and low-income countries. The majority of cases of AKI in high- and middle-income countries are hospital-acquired and most patients are elderly [5]. By contrast, there are few recent data on the incidence rates and causes of AKI from low-income countries, particularly from Africa. AKI in Africa is mostly community acquired, occurs in younger individuals, and is often due to a single cause [6]. 

In low-income countries, the diagnosis of AKI may not be made at all or may be delayed. Most small, rural health centers are not able to measure renal function. Even where these tests are available, doctors may not think of the possibility of AKI and may therefore not order kidney function tests, or the patients may not be able to afford these tests. Factors that influence survival are establishing the diagnosis, an understanding of the condition, and the availability of treatment. In one hospital in Ethiopia, 66 of 208 (32%) consecutive admissions did not have their kidney function measured [7]. In those patients in whom kidney function was measured, 20% had AKI. Even where AKI is diagnosed, dialysis may be unavailable, or the patient may not be able to afford dialysis. In a study from Burkina Faso, out of 84 cases of AKI who required dialysis, only 14 patients received this treatment [8]. The challenges of reducing the burden of AKI in low- and middle-income countries have been discussed [9]. These challenges are being addressed by the International Society of Nephrology in the AKI “0 by 25” initiative [10]. This initiative arises from the understanding that AKI is preventable and treatable and that many lives are being uselessly lost. 

## Methods 

We searched Pubmed for papers on acute kidney injury/acute renal failure in adults in Africa using a search filter [11] from 2000 to 2015. We combined this filter with a search for African countries and selected citations on adults. Data on the causes of AKI, the circumstances in which it occurred, treatment, and outcome was then analyzed. 

## Results 

### Incidence of AKI in Africa 

The incidence of AKI in hospitalized patients in Africa ranges from 0.3 to 1.9% in adults [12, 13] and 1 to 3% in children and adolescents [14, 15, 16, 17]. Most cases of AKI in Africa are community acquired in between 70 and 90% of cases [12, 15, 16, 17]. 

### Diagnosis of AKI in Africa 

There are relatively few recent studies of AKI in adults in sub-Saharan Africa, and few have used the Risk, Injury, Failure, Loss, End-Stage Renal Disease (RIFLE), Acute Kidney Injury Network (AKIN), or Kidney Disease: Improving Global Outcomes (KDIGO) criteria for AKI [12, 13, 18, 19, 20, 21, 22]. Most patients with AKI in Africa present late and between 56.2 and 80% of subjects had KDIGO AKI stage 3 [12]. 

### Demographics of patients with AKI in Africa (Table 1) 

Most adult patients with AKI in Africa are young, with a pooled mean age of 41.3 standard deviation (SD) 9.3 years with a male to female ratio of 1.2 : 1.0 [8, 12, 13, 18, 23, 24]. 

### Causes of AKI in Africa 

In Africa, medical causes account for between 65 and 80% of causes of AKI. This is followed by obstetric causes in 5 – 27% of cases and surgical causes in 2 – 24% of cases [8, 12, 13, 18, 23, 25, 26]. The major causes of AKI in urban areas include postsurgical and post-traumatic AKI in which is reported in between 15 and 37% of admissions to intensive care units [20, 24, 27, 28]. 

### Medical causes of AKI in Africa 

Sepsis is a major cause of AKI in Africa and is found in 26 – 40% of cases [12, 13, 18, 23, 24, 25]. Major causes of sepsis include cholera, other diarrheal illnesses, typhoid, and malaria. Massive intravascular hemolysis from infections and drugs remains an important cause of AKI in Africa, and this is more common in individuals with glucose 6 phosphate dehydrogenase (G6PD) deficiency. Nephrotoxins remain an important cause of AKI in Africa, and these include paraphenylene diamine, which is a hair dye [25], herbal remedies [29], and conventional medications. Another important cause of AKI in Africa is envenomation from snake bites [30]. AKI is a major complication of HIV infection, and the contributing factors of this were sepsis, volume depletion, and nephrotoxins [31]. 

### Pregnancy AKI 

Pregnancy-associated AKI is common in Africa, with an incidence of ~ 1 in 1,000 deliveries [12, 32]. Pregnancy-associated AKI accounts for between 5 and 27% of all causes of AKI [12, 33]. This is between 20 and 100 times more common than in high-income countries. The major causes of AKI in pregnancy are hemolysis, elevated liver enzymes, low platelet count (HELLP) syndrome, eclampsia or pre-eclampsia, and peripartum hemorrhage [12]. 

### AKI and obstructive uropathy 

Obstructive uropathy is an important but little-reported cause of AKI in Africa. Between 6 and 20% of cases of AKI in Africa had an obstructive uropathy [12, 23, 25], and the major causes were pelvic cancer and kidney stones [12, 25], carcinoma of the cervix, endometrium, and ovary in women and carcinoma of the prostate in men [12]. 

### AKI in the intensive care unit 

In urban areas with good hospitals there are an increasing number of reports of AKI requiring intensive care. The major causes of AKI in this setting include surgery, trauma, and burns. In this setting, the incidence of AKI ranged from 13 to 35% and carried a mortality of between 50 and 70%, and this is similar to reports from developed countries [20, 24, 27, 28]. 

### Treatment of AKI 

In many parts of sub-Saharan Africa, individuals with AKI live in rural areas with no access to good health care. Dialysis is only available in specialist hospitals. In the reported studies, between 17 and 94% of patients who needed dialysis received this [8, 12, 13, 18, 21, 23, 25, 26]. Hemodialysis is more readily available than peritoneal dialysis. 

### Outcome of AKI 

The mortality of AKI in adults in Africa ranged from 11.5 to 43.5% [8, 12, 13, 18, 23, 25, 26, 33]. There are no data on follow-up kidney function. 

## Discussion 

The reported incidence of AKI in hospitalized adults in Africa ranges from 0.3 to 1.9% of all admissions [12, 13]. This is much lower than in high- and upper middle-income countries where the pooled incidence rate of AKI in hospitalized patients was 21.6% in adults (95% CI, 19.3 to 24.1) [5]. The most likely explanation for this is that renal function is not measured in most inpatients in Africa. In one study in Ethiopia, where renal function was measured prospectively, AKI diagnosed by the AKIN criteria was found in 20% of medical inpatients who had renal function tested [7]. By contrast, with the pattern in high- and middle-income countries where most cases of AKI are hospital acquired [5], most cases of AKI in Africa are community acquired in between 70 and 90% of cases [12]. More studies in Africa are now using the RIFLE, AKIN, and KDIGO criteria for the diagnosis of AKI, and this will make future comparisons of etiology, severity, and outcome of AKI easier [12, 13, 18, 20, 33]. Most patients with AKI in Africa are young, with a weighted mean age of 41 years. By contrast, patients in high- and middle-income countries are older, with a pooled mean age of 60.6 (range 23.5 – 80.3). 

The major causes of AKI in Africa are infections, diarrheal illnesses, and pregnancy associated kidney problems. Most patients with AKI are young and were previously well. Some causes of AKI in Africa are more common than those seen in developed countries. Thus, sepsis, hemolysis, pregnancy complications, and nephrotoxins are all common causes of AKI in Africa [12, 13, 23, 25, 33]. Pregnancy associated AKI in Africa is 20 – 100 times more common than in high-income countries [34]. The major causes of AKI in pregnancy are HELLP syndrome, eclampsia or pre-eclampsia, and peripartum hemorrhage [12, 33]. These are complications of hypertensive diseases of pregnancy, which are common in Africa [35, 36]. There are now more reports of AKI secondary to obstructive uropathy. The causes include pelvic malignancies and renal and ureteric calculi [12, 25]. 

In urban areas of Africa where medical services are well developed, more AKI develops in hospital and in intensive care units. Patients admitted to intensive care units are older, have more severe disease, and a higher mortality than patients with community-acquired AKI [20, 27, 28]. Hemodialysis is only available in selected centers in Africa and mostly has to be paid for by the patient. In many parts of sub-Saharan Africa, most individuals with AKI live in rural areas with no access to good health care or dialysis. Most renal centers offer hemodialysis [12, 13, 18, 23, 25, 26, 33], and some offer peritoneal dialysis as well [25, 26]. The ISN “0 by 25” AKI vision of campaigning for treatment of AKI for all patients should lead to an improvement in the care of patients with AKI in Africa [10]. Where it is available, the outcome of treatment for AKI in Africa is good, with a survival rate of in excess of 60% 

## Conflict of interest 

None declared. 

**Table 1.** AKI studies in adults in Africa.

Study	Country	No of subjects	M/F	Age (mean ± SD)
Arogundade et al. 2007 [23]	Nigeria	46	34/12	38.2 ± 16.3
Kaballo et al. 2007 [25]	Sudan	89	57/32	39 ± 19.4
Lengani et al. 2010 [8]	Burkina Faso	121	75/46	38.6 ± 16.
Soliman et al. 2011 [26]	Egypt	51	29/22	48
Okunola et al. 2012 [33]	Nigeria	45	24/21	33.7 ± 10.1
Chijioke et al. 2012** [18]	Nigeria	138	58/80	29.4 ± 11.9
Emem-Chioma et al. 2013 [13]	Nigeria	62	34/28	41.3 ± 18.5
Okyere et al. 2015 [12]	Ghana	149	66/83	median 37 range: 14 to 88 years*

*Age given as median (range); **Data only on patients who received dialysis.
